# Abscisic Acid Signaling: Thermal Stability Shift Assays as Tool to Analyze Hormone Perception and Signal Transduction

**DOI:** 10.1371/journal.pone.0047857

**Published:** 2012-10-24

**Authors:** Fen-Fen Soon, Kelly M. Suino-Powell, Jun Li, Eu-Leong Yong, H. Eric Xu, Karsten Melcher

**Affiliations:** 1 Laboratory of Structural Sciences, Van Andel Research Institute, N.E., Grand Rapids, Michigan, United States of America; 2 Department of Obstetrics and Gynecology, National University Hospital, Yong Loo Lin School of Medicine, National University of Singapore, Singapore, Singapore; 3 State Key Laboratory of Drug Research, VARI-SIMM Center, Center for Structure and Function of Drug Targets, Shanghai Institute of Materia Medica, Shanghai Institutes for Biological Sciences, Chinese Academy of Sciences, Shanghai, People's Republic of China; Institute of Molecular and Cell Biology, Singapore

## Abstract

Abscisic acid (ABA) is a plant hormone that plays important roles in growth and development. ABA is also the central regulator to protect plants against abiotic stresses, such as drought, high salinity, and adverse temperatures, and ABA signaling is therefore a promising biotechnological target for the generation of crops with increased stress resistance. Recently, a core signal transduction pathway has been established, in which ABA receptors, type 2C protein phosphatases, and AMPK-related protein kinases control the regulation of transcription factors, ion channels, and enzymes. Here we use a simple protein thermal stability shift assay to independently validate key aspects of this pathway and to demonstrate the usefulness of this technique to detect and characterize very weak (Kd ≥50 µM) interactions between receptors and physiological and synthetic agonists, to determine and analyze protein-protein interactions, and to screen small molecule inhibitors.

## Introduction

Many signal transduction pathways are regulated by small molecules that bind to and modulate the activity of receptors and signaling enzymes. The protein thermal (stability) shift assay (TSA), also known as differential scanning fluorimetry (DSF) or thermofluor assay, is an increasingly popular method to identify small molecule ligands, as well as protein-stabilizing buffers and additives [1], [2], [3]. This method is based on changes in the thermostability of proteins upon binding of specific ligands or changes in the protein environment. As proteins are subjected to increasing temperature, their Gibbs free energy of unfolding decreases and eventually causes the native globular structure to become thermodynamically unstable and unfolding occurs [4]. The preferential binding of specific ligands, or non-specific interacting ingredients like salts, to the native form contributes to the free energy and increases protein stability and heat tolerance. Hence, measuring of the melting temperature (Tm, which is the midpoint of the transition from native to unfolded state) provides information both on the thermal stability of a protein as well as on conditions and specific ligands that promote stability changes.

TSA monitors the thermal unfolding of proteins with the use of environmentally sensitive dyes such as SYPRO Orange. The dye is quenched in aqueous surroundings and only fluoresces as the environment’s hydrophobicity increases. As the protein unfolds, the hydrophobic core residues become exposed to the dye and the resulting increase in fluorescence can be detected and recorded. With further increased temperature, the affinity between dye and hydrophobic residues decreases and hydrophobic residues become buried by protein aggregation, resulting in a decrease of fluorescence [1], [3]. A major advantage of TSA is that it is compatible with standard real-time PCR thermocyclers and can be performed in 96 or 384-well formats. Although other biophysical techniques such as isothermal calorimetry (ITC), differential scanning calorimetry (DSC), and dynamic light scattering (DLS) have been developed to assess ligand binding and protein integrity, these procedures generally require the use of a larger amount of protein and lack throughput capability. Hence TSA, which has been developed and promoted as a high throughput-screening platform for the identification of stabilizing conditions and ligands, has received much attention and has been more widely applied in recent years [5], [6].

**Figure 1:**
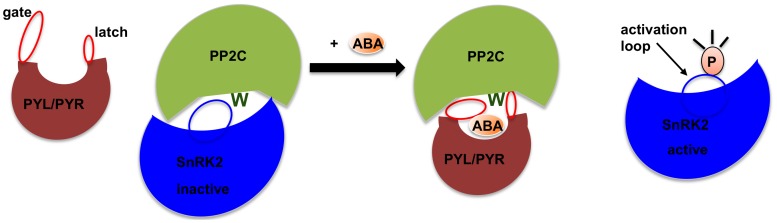
Cartoon presentation of the ABA core signaling pathway. In the absence of ABA, PYR/PYL receptors are in an “open” conformation with the “gate” loop facing away from the ligand-binding pocket and the “latch” loop. PP2Cs are active and bind to and inhibit SnRK2s by dephosphorylation of the activation loop as well as by allosteric inhibition. ABA-binding induces a conformation change in the receptors which causes the “gate” loop to swing in a closed position and a conserved tryptophan residue (represented as dark green W) on the PP2C surface “locks” the ligand in the pocket, forming a catalytically inactive receptor/ligand/phosphatase complex. PP2C inhibition allows SnRK2 activation by activation loop phosphorylation, which enables SnRK2s to transduce the ABA signal by phosphorylating downstream effectors.

In this study, TSA was employed to examine a well-characterized plant signaling pathway – the abscisic acid or ABA signaling pathway. ABA is a phytohormone that plays a pivotal role in plant growth and coordinates responses to adverse environmental conditions such as drought and salinity [7], [8], [9], [10]. Genetic and structural studies have unraveled a core signaling pathway that is regulated by phosphorylation-dephosphorylation events, which are mediated by stress response phosphatases and kinases. The presence of ABA is perceived by receptors termed PYR/PYL/RCARs, which, upon ligand binding, undergo a conformational change that enables them to interact with and inhibit type 2C protein phosphatases (PP2Cs) [11], [12], [13]. In their active state, PP2Cs bind and inhibit Snf1-related kinase 2 proteins (SnRK2s), both by blocking access to the kinase active cleft and by activation loop dephosphorylation [14]. ABA-mediated PP2C inhibition therefore releases SnRK2s from their inactive state and allows them to phosphorylate and activate downstream effectors to regulate stress-responsive programs [15], [16], [17]. Fig. 1 shows a cartoon presentation of the core ABA signaling pathway. In this report we have used the well-established ABA pathway as a model to test TSA on its ability to analyze signaling processes. We demonstrate that the data generated by TSA agree well with published biochemical and structural studies and that the approaches used may be applicable to the interrogation of other signaling pathways.

**Figure 2:**
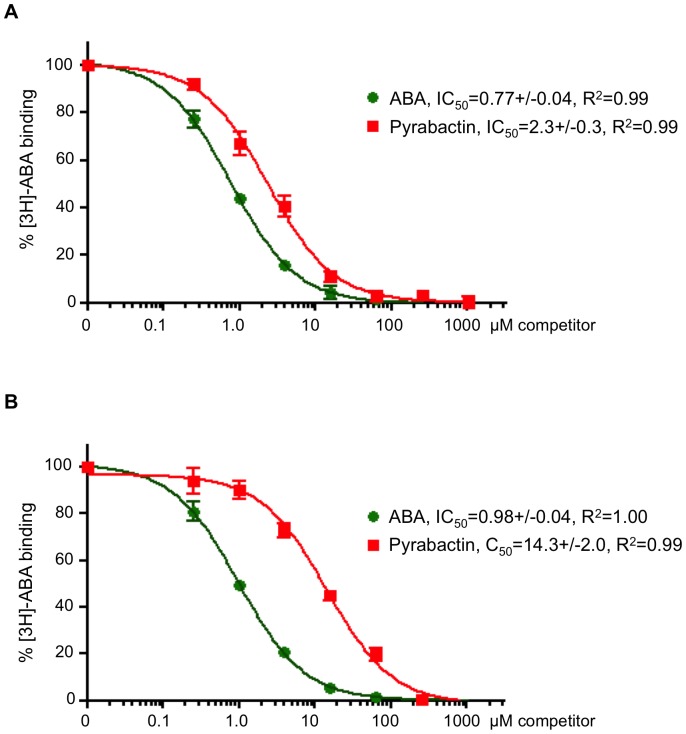
PYL1/PP2C and PYL2/PP2C radioligand competition assays. Competition of the interactions between ^3^H-ABA and the PYL1/HAB1 (**A**) and PYL2/HAB1 (**B**) coreceptor complexes by ABA and pyrabactin (n = 3, error bars represent s.d.).

## Materials and Methods

### Protein Preparation

PYL1 (residues 36–211), PYL2 (residues 14–188), HAB1 (residues 172–511), ABI2 (residues 101–423), and full length SnRK2.3 and SnRK2.6 were expressed as recombinant proteins in *E. coli* and purified as described previously [18], [19].

**Figure 3:**
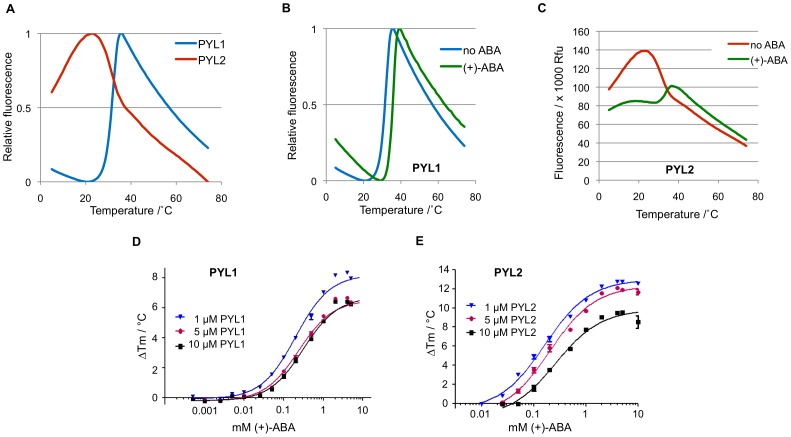
ABA-induced stabilization of PYL1 and PYL2 in a dose-dependent manner. (**A**) Melt curves of apo-PYL1 and -PYL2. (**B**) Melt profile of PYL1 in the presence of high concentration of (+)-ABA. Shift in the melt curve upon addition of 500 µM of (+)-ABA suggests a stabilization effect due to binding. (**C**) Melting profile of PYL2 in the presence of 25 µM (+)-ABA. Initial fluorescence was reduced and a two-state transition was observed. (**D+ E**) (+)-ABA/ΔTm dose-response curves for PYL1 (**D**) and PYL2 (**E**).

MBP-SnRK2.3 was constructed as a fusion protein with MBP tagged to the N-terminus of full-length SnRK2.3 with a NAAAEF linker. Both MBP and MBP-SnRK2.3 were expressed in *E. coli* BL21 (DE3) and purified from 2 l of cells. Cells were lysed by sonication and the cleared lysate was loaded onto a 20 ml amylose column and washed with 100 ml buffer A (20 mM Tris pH 8.0, 200 mM NaCl, 10% glycerol) before eluting in 20 mM Tris pH 8.0, 200 mM NaCl, 10 mM maltose and 10% glycerol. The fusion protein was further purified by Superdex 200 gel filtration chromatography in 25 mM Tris, pH 8.0, 200 mM ammonium acetate, 1 mM dithiotreitol, and 1 mM EDTA. Only the monomeric fractions from the gel filtration columns were used for TSA experiments.

**Figure 4:**
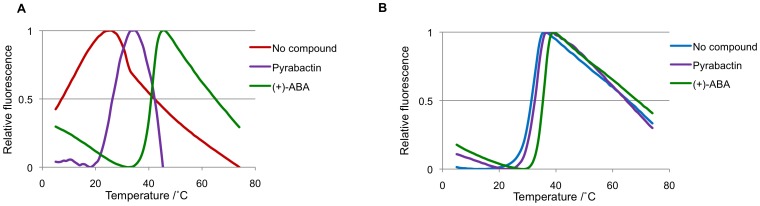
PYL receptor binding of ABA and pyrabactin. (**A + B**) Melt curves of PYL2 (**A**) and PYL1 (**B**) in the absence and presence of 500 µM of (+)-ABA and pyrabactin.

### Thermal Stability Shift Assay (TSA)

All reactions were set up in final volumes of 10 µl in 96-well plates with either 10× (1∶500 dilution of stock; for total protein concentrations of 10 µM or less) or 20× SYPRO Orange (Invitrogen) and incubated with compounds on ice for 30 mins. Reactions for the detection of ternary PYL-ABA-PP2C complexes included 500 µM of (+)-ABA. Compounds from four kinase inhibitor libraries (Calbiochem I, II, III, Tocriscreen Inhibitor Toolbox) were screened at 50 µM for Tm increases of 5 µM MBP-tagged SnRK2.3 and untagged SnRK2.3 and SnRK2.6. Total DMSO concentration was restricted to 2% or less.

**Figure 5:**
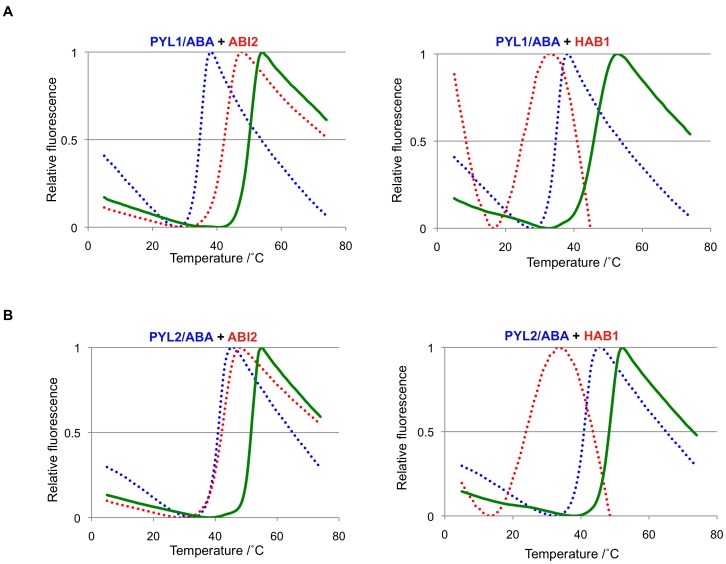
Formation of ternary receptor/ligand-phosphatase complexes. Both the PYL1 and PYL2 receptors were able to interact with the HAB1 and ABI2 phosphatases to form complexes in the presence of (+)-ABA. Melt curves of ligand-bound receptors (dashed blue lines), PP2Cs (dashed red lines), and receptor-PP2C complexes (solid green lines). Receptors and PP2C were at 10 µM, (+)-ABA at 500 µM. (**A**) PYL1 with ABI2 (left) and HAB1 (right). (**B**) PYL2 with ABI2 (left) and HAB1 (right).

**Figure 6:**
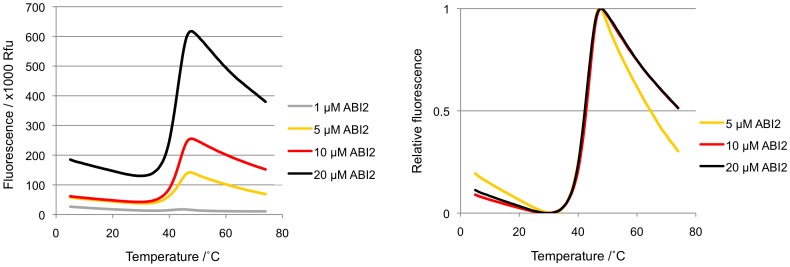
ABI2 melt curves at different ABI2 concentrations. The normalized melt curves are superimposed on each other in the right panel. Note that fluorescence increases with protein concentration, while the Tm is independent of ABI2 concentration.

**Figure 7:**
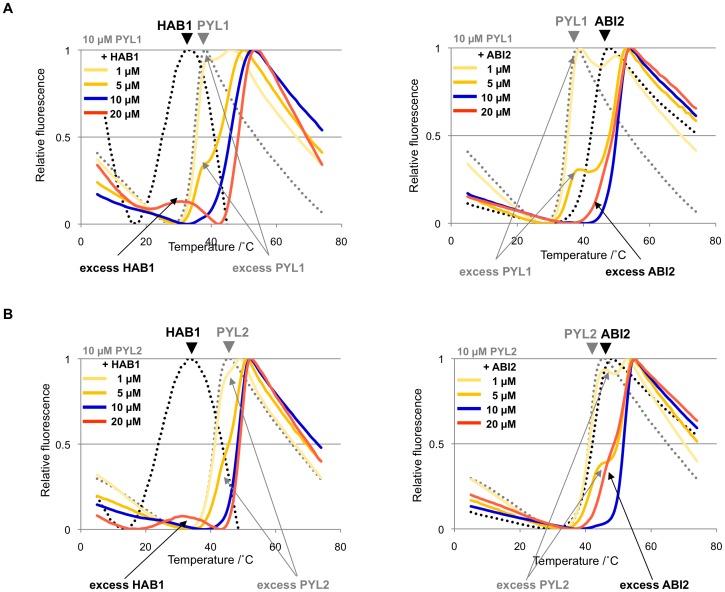
Stoichiometry of receptor/ligand-phosphatase complexes. Transition curves of ABA-bound PYL1 and PYL2 in the presence of increasing amounts of HAB1 and ABI1 PP2Cs. The melt curves for receptors in the absence of PP2Cs (dashed grey lines ) and PP2Cs in the absence of receptors (dashed black lines ) are shown as references. The positions of uncomplexed, excess receptors and PP2Cs are indicated by arrows. Each panel shows titrations of 10 µM receptor with PP2Cs at 1 µM (pale yellow line), 5 µM (yellow line), 10 µM (blue line) and 20 µM (orange line) PP2C. (**A**) PYL1/ABA titrated with HAB1 (left) and ABI2 (right). (**B**) PYL2 titrated with HAB1 (left) and ABI1 (right).

Thermal melting experiments were carried out using the StepOnePlus™ Real-Time PCR System (Applied Biosystems) melt curve program with a ramp rate of 1°C and temperature range of 15°C to 85°C for the kinase inhibitor screen and 4°C to 74°C for all other experiments. All experiments were performed in triplicates except for the screen against the kinase inhibitor libraries.

**Figure 8:**
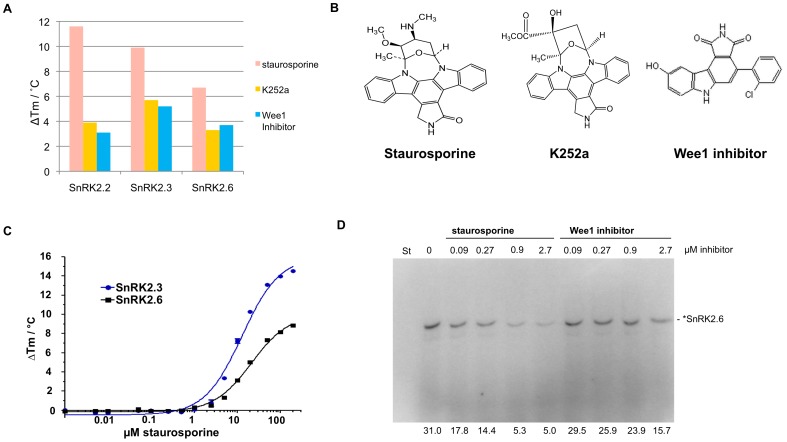
Increase in thermal stability of SnRK2s upon binding of kinase inhibitors. (**A**) Incubation with staurosporine, Wee1 inhibitor, and K-252a shifted the Tm of all three ABA-signaling SnRK2s. (**B**) Chemical structure of the kinase inhibitors that stabilized and increased the Tm of SnRK2.2, 2.3, and 2.6 kinases by at least 3°C. (**C**) Dose-dependent stabilization of 5 µM SnRK2.3 and SnRK2.6 by staurosporine. (**D**) Inhibition of SnRK2.6 autophosphorylation by staurosporine and Wee1 inhibitor in a radioactive kinase assay. 100 nM SnRK2.6 were incubated with [^32^P]-γ-ATP and increasing concentrations of kinase inhibitors. Reactions were separated by SDS PAGE and dried gels exposed to PhosphorImager screens. A densitometric quantitation of autophosphorylation bands is shown below the autoradiogram.

### Analysis of TSA Data

Data analysis was performed according to the protocol described by Niesen *et al.* [*1*]. Melting temperatures (Tm) were calculated by fitting the sigmoidal melt curve to the Boltzmann equation using GraphPad Prism, with R^2^ values of >0.99. ΔTm is the difference in Tm values calculated for reactions with and without compounds. For titration data, the ΔTm values were averaged and plotted against concentration of compound and fitted to a single binding-site model.

**Figure 9:**
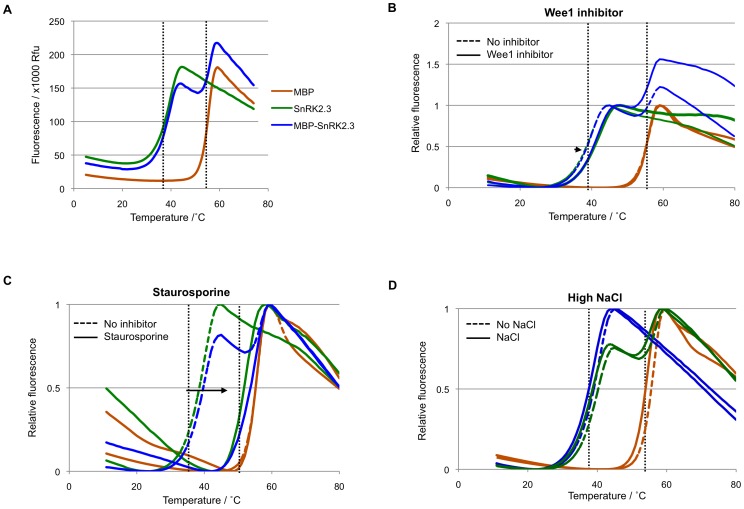
SnRK2 kinase inhibitors affect the thermostability of the SnRK2.3 moiety, but not the MBP moiety, of a MBP-SnRK2.3 fusion protein. (**A**) Melting profiles of SnRK2.3 (green), MBP (brown), and a MBP-SnR2.3 fusion protein (blue). The fusion protein underwent two separate unfolding transitions: the transition at lower temperature corresponds to that of the kinase while the unfolding at the higher temperature corresponds to that of the more stable MBP tag. (**B–D**) Melt curves of SnRK2.3, MBP, and the MBP-SnRK2.3 fusion protein in the presence of Wee1 inhibitor (**B**), staurosporine (**C**), and 500 mM NaCl (**D**). The color code is the same as in (A), with dashed lines representing the melt curves in the absence of inhibitors and solid lines those with inhibitors. Dotted black lines indicate the Tm of SnRK2.3 and MBP in the absence of inhibitors.

**Figure 10:**
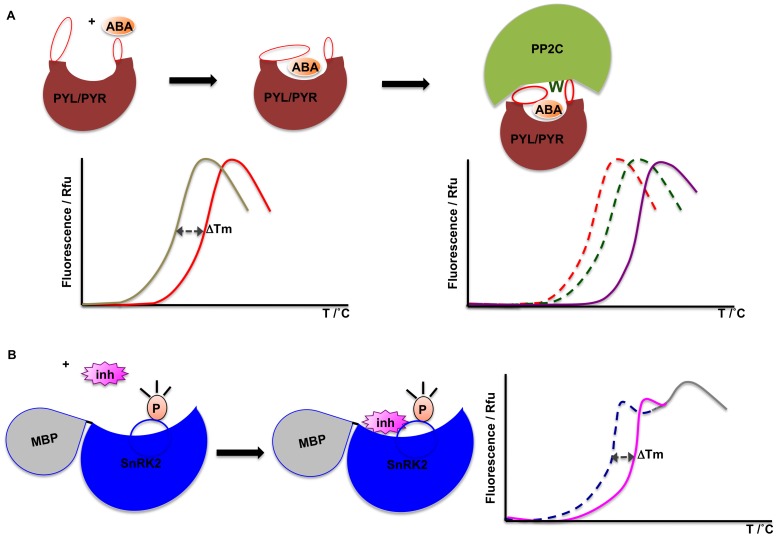
Schematic representation of thermostability profiles corresponding to the various aspects of the signaling pathway. (**A**) Stabilization of receptor by ligand-binding is demonstrated by the shift in the melt curve, and ligand-induced protein-protein interaction results in the formation of a more stable complex that unfolds cooperatively with a Tm higher than that of both individual proteins. (**B**) A SnRK2-MBP fusion protein exhibits a bi-phasic melt curve, of which only the transition curve corresponding to untagged SnRK2 shifts in the presence of SnRK2 inhibitors.

### Radio-ligand Binding Competition Assay

300 µg of yttrium silicate copper-chelating scintillation proximity assay (SPA) beads (GE Healthcare) were incubated with a large excess of H6GST–PYL1 or –PYL2 (∼20 µM) in a buffer of 50 mM MOPS, pH 7.4, 50 mM NaF, 50 mM CHAPS, and 0.1 mg ml^−1^ bovine serum albumin for 80 min shaking on ice. H6GST–PYL bound to SPA beads was separated from free H6GST–PYL by centrifugation at 5,200 g for 30 s. Bead pellets were washed with 1 ml of the same buffer, then resuspended in 120 µl of the buffer supplemented with 450 nM ^3^H-labelled ABA (GE Healthcare), 10 µM HAB1, and the indicated amounts of unlabelled (+)-ABA and pyrabactin, and then incubated shaking for 1 h at room temperature. ^3^H-ABA bound to the SPA beads was quantitated by liquid scintillation counting.

### Kinase Activity Assays

100 nM H6GST-SnRK2 kinase aliquots were incubated with inhibitors at indicated concentrations in 25 mM Tris, pH 7.3, 5 mM MgCl_2_, 1 mM DTT, 100 µM EGTA, 100 nM unlabeled ATP and 0.5 µCi [^32^P]-γATP for 35 min at room temperature in a total volume of 15 µl. Reactions were terminated by addition of SDS sample buffer and subjected to Tricine SDS-PAGE. Gels were dried and subjected to autoradiography using a FLA-5000 phosphor imager (Fuji).

## Results

### Stabilization of PYL Receptors Upon Ligand Binding

*Arabidopsis thaliana* utilizes 13 RYR/PYL/RCAR ABA receptors that differ in ABA affinity, expression, and response to synthetic ABA agonists. Dimeric receptors, such as PYL1 and PYL2, bind ABA with very low affinities, estimated at 50–350 µM [20], [21]. However, ABA affinity increases by one to two orders of magnitude in the presence of ABA-signaling PP2Cs, which can function as ABA coreceptors [18], [20], [21] (see Figure 1). The low ligand affinity of dimeric ABA receptors in the absence of their PP2C coreceptors presents a formidable challenge for both the detection and quantification of ligand binding. We were able to use radioligand competition assays to estimate ABA affinities for PYL1/PP2C and PYL2/PP2C coreceptor complexes to be ∼1 µM (Fig. 2 and Materials and Methods). In contrast, the low-affinity binding of ^3^H-ABA to either apo receptor was too transient to be detectable by radioligand binding. We therefore tested whether TSA would allow to analyze such a weak interaction.

In order to determine the effect of ABA on the thermostability of PYL1 and PYL2, we first recorded the melt curves of the two apo ABA receptors. The PYL1 melting profile resembled the typical sigmoidal curve with a two-state transition that allowed the derivation of the Tm. In contrast, apo-PYL2 did not display a conventional melt curve despite the structural and biochemical similarity of both receptors (Fig. 3A). The presence of high concentrations of the physiological ABA isomer (+)-ABA appeared to stabilize PYL1 and increased the Tm, supporting the findings that PYL1 binds ABA (Fig. 3B). Interestingly, PYL2 displayed a melt curve similar to that of PYL1 upon the addition of 25 µM (+)-ABA, even though a clear Tm derivation was not possible for the apo form (Fig. 3C).

To study the effects of ABA concentration on the Tm of both PYL1 and PYL2, increasing amounts of (+)-ABA were added to the receptor proteins. Theoretical TSA dose-response curves show a sigmoidal dependence of ΔTm on ligand concentration until, upon ligand saturation of the protein, further increases in ligand concentration result in linear increases in ΔTm up to the solubility limit of the ligand. For an ideal curve, binding constants (K_D_) and stoichiometry can be determined by curve fitting as described [22], [23], [24]. As shown in Fig. 3D and 3E, both PYL1 and PYL2 display characteristic sigmoidal ABA dose response curves, but lack the linear increase phase due to limited ABA solubility, which precludes an accurate K_D_ determination. Tm increases were not detectable at 1∶1 stochiometric receptor:ABA ratios, but rather required excess ABA concentration of 50 µM (PYL1) and 25 µM (PYL2) (Fig. 3D+3E). Since detectable Tm increases at stochiometric or super-stochiometric ligand:receptor ratios require ligand concentrations approaching K_D_ [22], these data are consistent with ABA binding constants ≥50 µM, as has been reported for both receptors [20], [21].

The PYR/PYL ABA receptors were identified using a chemical genetic screen that exploited the receptor subtype-selectivity of a synthetic ABA receptor agonist, pyrabactin [12]. Pyrabactin is an efficient agonist for the PYR1 receptor and a weaker agonist for PYL1 [12]. In contrast, pyrabactin functions predominantly as antagonist (mixed antagonist/weak agonist) for the PYL2 receptor by adopting a non-productive conformation in the PYL2 ligand–binding pocket. Pyrabactin thus competes with ABA for PYL2 binding, but most of the pyrabactin-bound PYL2 fails to interact with PP2Cs [19], [25], [26], [27]. As expected, PP2Cs such as HAB1 therefore poorly stabilize the binding of pyrabactin to PYL2 (Fig. 2). TSA allowed us to also analyze pyrabactin-binding to apo PYL2. The presence of pyrabactin, similar to that of ABA, allowed PYL2 to undergo a normal melting transition and to calculate a Tm, which is lower than that in the presence of ABA (Fig. 4), suggesting that pyrabactin directly binds PYL2, but with an affinity that is lower than that of ABA.

### Ligand-induced Protein-protein Interaction

ABA-bound PYL receptors initiate the signaling cascade by binding to PP2Cs. Binding inhibits the PP2Cs [11], [12], [13], [18], [20], [21], thereby allowing the SnRK2s to auto-activate by activation loop autophosphorylation [14], [28], [29], [30], [31], [32]. To investigate ABA-dependent PYL–PP2C interactions, increasing concentrations of two ABA-signaling PP2Cs, ABI2 and HAB1, were mixed with either 10 µM PYL1 or PYL2 in the absence and presence of ABA. The resulting profiles, as seen in Fig. 5A and B, show a smooth melt curve with a Tm that was higher than those of PYL/ABA or PP2C alone (see also Fig. 3 and Fig. 6), indicating the formation of a more stable complex that unfolds cooperatively when subjected to thermal melting. The PP2C titration also revealed the stoichiometry of the complexes (Fig. 7). At 1 and 5 µM of both ABI2 and HAB1, the melt curves consisted of two peaks, one of which corresponded to the more stable PYL–PP2C complex and the other to excess ABA-bound receptor (Fig. 7 pale yellow and yellow profiles). A biphasic melting profile was also observed with 20 µM of PP2Cs, reflecting the presence of the complex and excess apo-phosphatase (Fig. 7 orange profiles). In contrast, addition of 10 µM phosphatase to 10 µM receptor yielded smooth single-peak profiles (Fig. 5 and Fig. 7 blue profiles). Thus, melt curves of receptor–PP2C complexes formed at varying receptor:PP2C ratios demonstrated that ABA-induced receptor–phosphatase complex formations occur at 1∶1 molar ratio, consistent with the crystal structures of several ternary PYL–ABA–PP2C complexes [14], [18], [19], [20], [21], [33], [34].

### Identification of Novel SnRK2.6 Inhibitors

The activity of SnRK2s determines the expression of ABA-responsive genes by controlling the phosphorylation and activation of downstream factors such as the ABA box-binding factor family of basic leucine zipper transcription factors [35], [36], [37], [38]. Hence we used TSA to screen for kinase inhibitors that could possibly bind and interfere with the action of the three closely related SnRK2s that mediate ABA signaling: SnRK2.2, SnRK2.3 and SnRK2.6. Among the 3 kinases, SnRK2.2 and SnRK2.3 share a greater homology [39] and are involved in ABA signaling during seed germination and root growth [15], while SnRK2.6 regulates stomatal responses to ABA [16], [40], [41]. TSA analysis of the kinases reflects their evolutionary similarity as SnRK2.2 and SnRK2.3 have similar melting temperatures of 36.2°C and 38.3°C respectively, while SnRK2.6 is much more stable with a Tm of 49.0°C [28]. We screened approximately 300 commercially available kinase inhibitors at 50 µM concentration for compounds that stabilize all three kinases. Only three of the inhibitors increased the Tm of all three kinases by at least 3°C: staurosporine, the staurosporine analog K252a, and Wee1 inhibitor (Fig. 8A and B). Staurosporine, a broad-spectrum inhibitor of many kinases, including SnRK2s [31] and the mammalian SnRK2 homolog AMPK [42], increased the Tm of SnRK2.2 and SnRK2.3 by more than 9°C, and SnRK2.6 to a lesser extent of 6.7°C (Fig. 8A). Titration of staurosporine to 5 µM of SnRK2.3 and SnRK2.6 produced a noticeable increase in Tm at a minimum concentration of 2.5 µM (Fig. 8C). The greater ΔTm observed for SnRK2.3 suggests that the affinity of staurosporine is higher for SnRK2.3 than SnRK2.6 (see Discussion).

The other two identified inhibitors, the staurosporine analog K252a and Wee1 inhibitor, both increased the Tm of all three SnRK2s by more than 3°C (Fig. 8A). We tested staurosporine and Wee1 inhibitor in *in vitro* kinase assays to validate their inhibitory effects on SnRK2s. As shown in Fig. 8D, SnRK2.6 autophosphorylation was inhibited by both compounds. Note that the concentrations required for inhibition of 50% of the kinase activity (IC_50_) inversely correlate with ΔTm shifts, a general feature of protein kinase inhibitors [5]. Hence the *in vitro* assay verified the thermal stability results as compounds found to stabilize the kinase were able to effectively inhibit its activity with IC_50_ that inversely correlated with the extent of thermo-stabilization.

### A Protein Purification Tag can Function as Internal TSA Specificity Control

Protein tags are commonly used as purification aids and often improve protein solubility and behavior. Fig. 9A demonstrates that a fusion protein consisting of full length SnRK2.3 separated from an N-terminal bacterial maltose-binding protein (MBP) tag by a flexible linker (see Material and Methods) shows a biphasic melting profile, which resembled the profile of apo-SnRK2.3 superimposed with that of apo-MBP. This suggests that the two modular proteins of the fusion do not affect each other’s melting characteristics when separated by a linker. Moreover, the MBP tag might actually serve as internal specificity control to differentiate between specific inhibitors for SnRK2.3 and compounds or conditions that non-specifically affect the thermostability of proteins or interfere with SYPRO Orange fluorescence.

To further explore this possibility, we repeated TSA in the presence of staurosporine and Wee1 inhibitor. Fig. 9B shows the normalized melt curves of apo-SnRK2.3, apo-MBP and MBP-SnRK2.3 fusion protein incubated with Wee1 inhibitor. The Tm of apo-MBP protein remained unchanged in the presence of the compound, suggesting that it was unbound and unaffected by the inhibitor that increased the free energy of untagged SnRK2.3. MBP-SnRK2.3 once again exhibited a transition curve with combined profiles of untagged SnRK2.3 and MBP. The Tm shift of the kinase moiety in the fusion protein was comparable to that of the untagged form, and the position of the MBP-corresponding peak was unchanged. We therefore conclude that Wee1 inhibitor binds to and stabilizes the SnRK2.3 module of the MBP-fusion protein, indicating that this interaction is specific and can be detected reliably. The more potent inhibitor staurosporine increased the Tm of the kinase to a much greater extent, which caused the melting curve of the stabilized SnRK2.3 to partially overlap with that of MBP (Fig. 9C). This resulted in the formation of a single peak profile, which showed a slightly larger ΔTm than the untagged kinase. Nonetheless, the MBP-SnRK2.3 fusion protein clearly allowed detection of the stabilization effect of staurosporine and the ranking of the two inhibitors according to their binding affinities.

In contrast to the specific inhibitor effects, high salt concentrations affect the stability of many proteins. In the presence of 500 mM NaCl, the Tm of all three proteins were reduced by 1.5–1.6°C (Fig. 9D), indicating a destabilization effect. Importantly, both peaks of the MBP-SnRK2.3 fusion protein also showed shifts corresponding to those of their untagged counterparts (Fig. 9D). The movement of both peaks in MBP-SnRK2.3 indicates that the effect of high salt concentration on thermostability is not specific to SnRK2.3. Thus, a fusion tag like MBP can function as internal control to recognize specific protein binding events as well as to identify conditions that nonspecifically affect protein integrity.

## Discussion

TSA is typically used to identify conditions that increase protein stability and to identify small molecule ligands. Here we have used TSA to analyze and independently validate aspects of the core ABA signaling pathway. Specifically, we have studied three stages of the signaling mechanism: ligand perception by receptor, ligand-induced protein complex formation, and inhibition of the resulting kinase activation (Fig. 10).

Binding of ABA to the monomeric receptors PYL1 and PYL2 is extremely weak (K_D_≥50 µM), which makes detection by standard biochemical assays, such as radioligand-binding and surface plasmon resonance assays, either impossible or very challenging. The gold standard for analyzing weak protein-small molecule interactions is isothermal calorimetry (ITC), which provides detailed kinetic information on interactions. However, ITC requires large volumes of protein and ligand at very high concentrations (ideally protein at ≥10× K_D_ and ligand at a concentration ≥10× that of the protein) [43], [44], which often puts practical limits on the solubility of protein and ligand at very high K_D_. In contrast, TSA is routinely performed at ≤10 µl reaction volumes with proteins at low micromolar concentrations. It is easily and rapidly performed in medium throughput (96 well plates) in standard real-time thermocyclers. Titration experiments can, under optimal conditions, provide estimates of binding constants by curve fitting [22], [23], [24], although we strongly recommend to validate any binding constant measurements by alternative assays. Using TSA, we have clearly been able to detect the low-affinity binding of (+)-ABA to the two dimeric receptors PYL1 and PYL2, as well as binding of the selective synthetic ligand pyrabactin to PYL2, a receptor whose interaction with pyrabactin had been demonstrated to be predominantly antagonistic [19], [26], [27].

To our knowledge, the interaction of the PYL1 and PYL2 receptor proteins with the PP2Cs ABI2 and HAB1 is the first example for the use of TSA in identifying physiological protein-protein interactions. In these experiments, PYLs and PP2Cs formed complexes that were more stable than the individual proteins and that unfolded cooperatively with elevated Tm when subjected to thermal melting. Moreover, titrations of one of the binding partners resulted in formation of biphasic melting curves, with one phase overlapping with the melting curve of the complex and the other with that of the protein in excess. The titration TSA experiments therefore provided not only a better-controlled binding reaction, but also allowed accurate determination of binding stoichiometries, with results consistent with the crystal structures of these complexes. These analyses require that the individual proteins and the protein complexes exhibit distinct melting curves with only limited overlap. When these requirements are met, the TSA titration assay should be generally applicable to determine and analyze protein-protein interactions.

ABA-mediated PYL binding inhibits PP2Cs and thereby activates SnRK2 kinases. We have applied TSA to screen a panel of ∼300 commercially available kinase inhibitors for stabilization of 3 ABA-responsive SnRK2s: SnRK2.2, SnRK2.3 and SnRK2.6. Previous studies have shown a strong inverse quantitative correlation between ΔTm and IC_50_ values for kinase inhibition, which allows a ranking estimate of relative affinities based on ΔTm [5], [6], [45]. Generally, Tm shifts due to ligand binding are proportional to the affinity of the ligand for compounds whose binding causes similar enthalpy changes, as is the case for most inhibitors of protein kinases. TSA can therefore provide a medium- to high-throughput solution to not only screen, but also rank related compounds based on their relative ΔTm [1], [46]. To our surprise, we identified just three kinase inhibitors that increased the Tm of the SnRK2s by at least 3°C: staurosporine, the staurosporine analog K252a, and Wee1 inhibitor. Of these, the highly promiscuous kinase inhibitor staurosporine was previously shown to inhibit members of the SnRK2 family [31], [47]. We verified by *in vitro* kinase assays that Wee1 inhibitor and staurosporine inactivated SnRK2.3, and that the relative concentrations required for inhibition were reflected by the relative increases in protein thermostability.

Finally, we have demonstrated that fusion of SnRK2.3 to a stable, non-interacting protein tag can provide an internal selectivity control, thereby improving the quality of the screen output and increasing the confidence in the hits and conditions identified. This method works best when the two domains of the fusion protein differ greatly in their Tm and show discrete, non-overlapping transitions. In addition, this method may also prove to be beneficial for proteins that purify better with a tag. Many proteins are expressed as tagged fusion constructs to aid in purification and also to increase yield and solubility. An elimination of the need for tag removal would therefore shorten the protein purification process as well as enabling TSA analysis of proteins that require a tag for stability.

In conclusion, we have adapted a fast and relatively simple thermostability assay to analyze multiple aspects of a signaling pathway, including low-affinity ligand binding, detection and stoichiometry of protein-protein interactions, and internally-controlled compound screening. These experiments have provided insight into ABA signaling and may be applied to the analysis of other signal transduction pathways.
